# Predictors for malignancy risk in subcentimeter thyroid nodules categorized as atypia/follicular lesion of undetermined significance by fine needle aspiration

**DOI:** 10.1038/s41598-019-50597-z

**Published:** 2019-10-18

**Authors:** Jinhua Ding, Li Jiang, Jianjiang Fang, Yan Jiang, Ye Zhu, Tebo Hua, Yijie Yuan, Weizhu Wu

**Affiliations:** 1 Department of Breast and Thyroid Surgery, Ningbo Medical Center Lihuili Eastern Hospital/Taipei Medical, University Ningbo Medical Center, Ningbo, 315000 China; 2 Department of Emergency, Ningbo Medical Center Lihuili Eastern Hospital/Taipei Medical University Ningbo Medical Center, Ningbo, 315000 China; 3 Department of Ultrasonograpy, Ningbo Medical Center Lihuili Eastern Hospital/Taipei Medical University Ningbo Medical Center, Ningbo, 315000 China

**Keywords:** Cancer prevention, Risk factors

## Abstract

Little work has been done on the prediction of malignancy risk in patients with subcentimeter thyroid nodule (TN) categorized as atypia/follicular lesion of undetermined significance (AUS/FLUS). We performed a retrospective analysis on the medical records of subcentimeter TNs whose initial fine-needle aspiration (FNA) diagnosis was AUS/FLUS at our center between November 2013 and August 2018. Univariate analysis and multivariate logistic regression analysis were used to select independent factors associated with malignancy. Of the 324 patients who were classified as AUS/FLUS on initial FNA, 153 patients underwent surgical procedures and showed an associated malignancy rate of 45.10% (69/153). The malignancy rates in AUS/FLUS settings with and without repeat FNA were 38.30% (18/47), and 48.11% (51/106), respectively, p = 0.260. Multivariate logistic regression analysis revealed that age < 55 (OR 3.015, 95% CI 1.196–7.596), microcalcification (OR 9.162, 95% CI 3.332–25.916) and taller than wide shape (OR 10.785, 95% CI 4.108–28.319) were three independent predictors for malignancy. The malignancy rates in the patients with one or none of predictor and patients with two or three above predictors were 20.5% (17/83) and 74.3% (52/70), respectively, p < 0.001 (OR 11.216, 95% CI 5.266–23.885). In conclusion, our study showed that for subcentimeter TNs with AUS/FLUS category, patient’s age, taller than wide shape and microcalcification were three independent predictive factors for malignancy, which was helpful for decision-making of surgery or observation in such patient population.

## Introduction

To date, the Thyroid Imaging Reporting and Data System (TI-RADS) is considered as the main criteria for determining malignancy and are generally followed by radiologists and physicians in practice. Ultrasound guided fine needle aspiration (FNA) and Bethesda System for Reporting Thyroid Cytopathology (BSRTC) are considered as the most accurate and cost-effective methods for the diagnosis of thyroid nodules preoperatively, but the accuracy of FNA performance has varies among different nodules^1–4^. However, these categorization systems are established based on FNA cytology results that included data from nodules >1 cm.

BSRTC category system includes six categories: unsatisfactory, benign, atypia/follicular lesion of undetermined significance (AUS/FLUS), suspicious for follicular neoplasm/follicular neoplasm (sFN), suspicious for malignancy (sM), and malignancy. Among them, AUS/FLUS category is the most challenging due to its low malignancy risk. A few studies have evaluated the values of ultrasonography for prediction of malignancy in thyroid nodules with AUS/FLUS cytology, and demonstrated that taller than wide shape, microcalcification and growing fast were identified to be independent predictive factors for malignancy^5–9^. However, the patients included in those studies were from outside China, and the size of nodules was larger than 1 cm, because thyroid nodule <1 cm (also named as subcentimeter TN) was not routinely recommended for FNA in the American Thyroid Association (ATA) guideline^10^.

Subcentimeter TN with any suspicious ultrasound characteristic for malignancy is routinely recommended to undergo FNA in our center, if patients prefer to exclude thyroid malignancy or have high risk factors such as family history of thyroid cancer, radiation history and evidence of lymph node metastasis. For those subcentimeter TNs with the cytological diagnosis of AUS/FLUS, the further managements represent an ongoing challenge, because no research has been carried out to support any of the following managements: clinical observation, ultrasound follow-up, repeat FNA or surgery.

A wide range of 6–48% malignancy risk in specimens categorized as AUS/FLUS was described in previous studies^11–15^. However, for subcentimeter TNs with suspicious US features, the accurate malignancy rate is unclear, and whether there is any predictive factor for malignancy is uncertain. Therefore, our study aims to investigate the rate of malignancy in subcentimeter TNs categorized as AUS/FLUS, and further to explore predictive factors for malignancy in patients who underwent operation.

## Material and Methods

### Patients

After approval from the Institutional Ethics Committee, we retrospectively analyzed the data from thyroid aspirates at Ningbo Medical Center Lihuili Eastern Hospital between November 2013 and August 2018. Thyroid nodules with one or more of the following suspicious US characteristics: (1) poorly-defined margin; (2) taller than wide shape; and (3) microcalcification, were recommended to undergo FNA under US guidance regardless of the nodule size. For cases with multiple nodules, the specimen was obtained from the lesion that was suspicious for malignancy. Nodule size, location, composition, echogenicity and vascularity of the nodule were all evaluated. Repeat FNA was performed for a proportion of referral case in our institution. Written informed consent was obtained from every patient.

Exclusion criteria included: (1) patient with thyroid nodule ≥1 cm in greater diameter; (2) patient with a history of thyroid carcinoma; (3) patient with the evidence of neck lymph node metastasis; (4) patient without the final histopathology evaluation. Cystic or mixed nodules, isoechoic or hyperechoic nodules were also excluded from the study.

### Data collection

Demographic, clinical and biochemical data were collected, including age, gender, body-mass index (BMI), family history, nodule size, nodule laterality, free triiodothyroxine (FT3), free tetraiodothyroxine (FT4), anti-thyroid peroxidase antibody (TPOAb), anti-thyroglobulin antibody (TGAb), thyroid stimulating hormone (TSH), glucose, TC (total cholesterol), TG (triglyceride), HDL (high density lipoprotein), LDL (low density lipoprotein). US characteristics of the nodules were also recorded: (1) number and size of nodules, (2) echogenicity (referring homogenicity or inhomogenicity), (3) shape, (4) margin; (5) microcalcification; (6) intranodular central flow.

### Statistical analysis

T test for continuous variables, Chi-squared test or Fisher’s exact test for categorical variables were used to detect predictors for malignancy in univariate analysis. Then, multivariate logistic regression analysis, including all variables from univariate analysis that were associated with malignancy, was performed to test factors’ independence. P value of <0.05 was considered to have statistical significance; hazard ratio (HR) and 95% confidence intervals (CI) were also calculated. Statistical tests were two-sided, and analyses were performed using SPSS v. 20.0 Software (SPSS, Chicago, IL, http://www.spss.com).

## Results

### Patient characteristics

**Figure 1:**
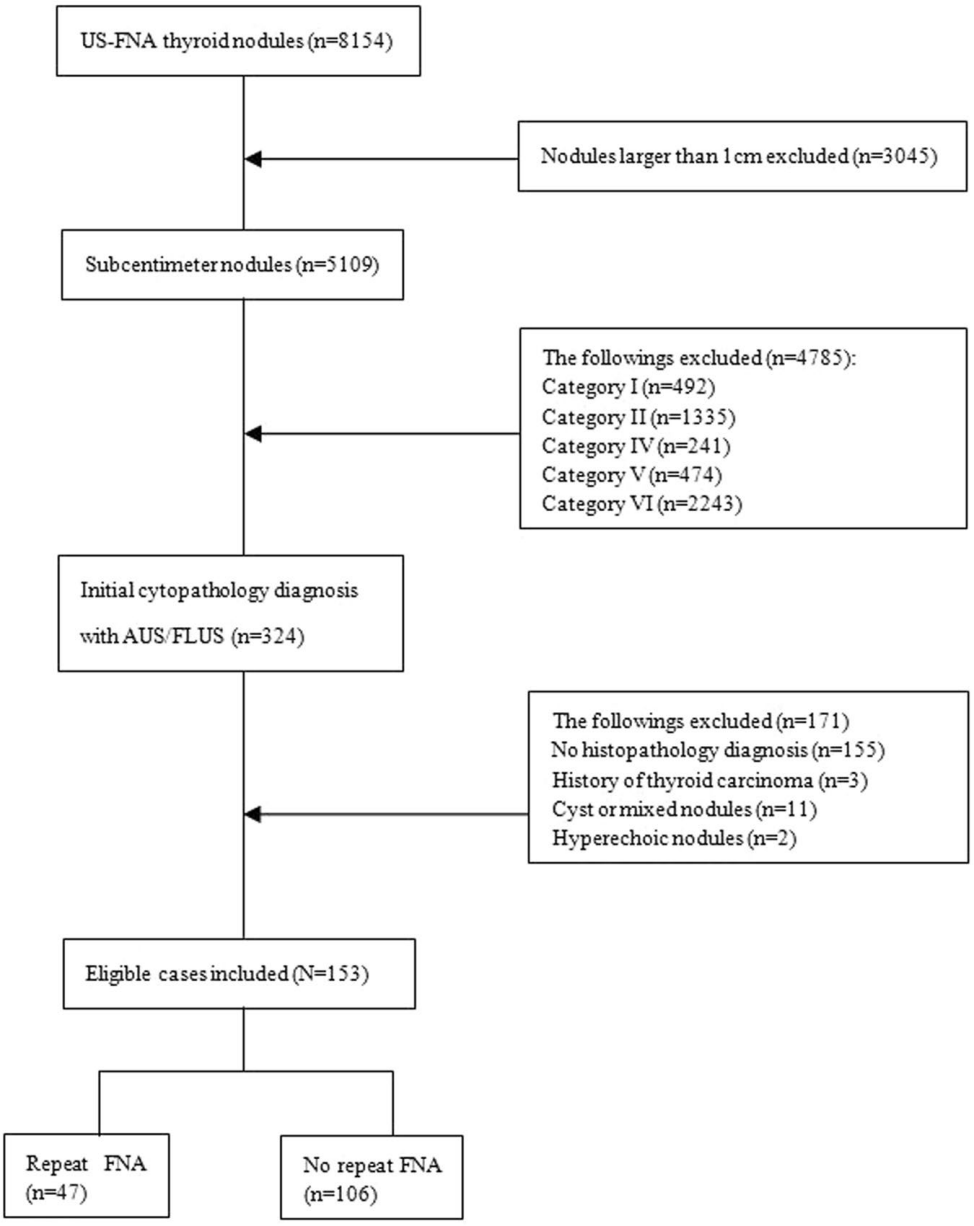
Flow chart of this study.

During the study period, a total of 8154 thyroid FNAs (including 3045 TNs ≥1 cm in greater diameter and 5109 subcentimeter TNs) were performed in our center. Among 5109 subcentimeter TNs, 324 (6.34%) specimens aspirated were interpreted as AUS/FLUS. Finally, 153 (47.22%) patients underwent subsequent surgical intervention, and included in our study (Fig. 1).

Of the 153 patients, there were 118 (77.12%) females and 35 (22.88%) males, and the mean age was 47.0 ± 13.2 year. Final histopathology yielded 52 (33.99%) benign thyroid goiters, 23 (15.03%) thyroiditis, 9 (5.88%) adenomas, and 69 (45.10%) malignancies, including 62 papillary thyroid carcinomas and 7 follicular thyroid carcinomas. Female and age <55 year are associated with the higher rate of malignancy. The baseline patient characteristics on benign and malignancy groups are compared in detail (Table 1).

**Table 1:** Baseline characteristics of patients with AUS/FLUS diagnosis.

Variable	All patients (n = 153)	Malignancy (n = 69)	Benign (n = 84)	p value
Age (≤55)	114 (74.51)	58 (84.06)	56 (66.67)	0.014
Gender (male)	35 (22.88)	10 (14.49)	25 (29.76)	0.005
BMI (≤25 kg/m^2^)	108 (70.59)	50 (72.46)	58 (69.05)	0.644
FT3 (pmol/L)	4.48 ± 0.52	4.43 ± 0.52	4.52 ± 0.53	0.809
TSH (mIU/L)	2.00 ± 1.33	1.95 ± 1.30	2.06 ± 1.37	0.857
FT4 (pmol/L)	15.96 ± 2.19	15.82 ± 2.17	16.13 ± 2.21	0.123
TGAb (>60 IU/ml)	49 (32.03)	21 (30.43)	28 (33.33)	0.702
TPOAb (>60 IU/ml)	60 (39.22)	31 (44.93)	29 (34.52)	0.190
Glucose (mmol/L)	5.47 ± 1.08	5.57 ± 1.08	5.37 ± 1.08	0.146
TC (mmol/L)	4.95 ± 0.92	4.96 ± 0.92	4.95 ± 0.92	0.823
TG (mmol/L)	1.49 ± 1.21	1.60 ± 1.40	1.39 ± 1.01	0.193
HDL (mmol/L)	1.38 ± 0.31	1.37 ± 0.30	1.39 ± 0.32	0.821
LDL (mmol/L)	3.01 ± 0.72	3.00 ± 0.70	3.01 ± 0.74	0.909
Nodule on the left	73 (47.71)	33 (47.83)	40 (47.62)	0.980
Nodule size (≤0.5 cm)	21 (13.73%)	8 (11.59)	13 (15.48)	0.487

AUS/FLUS: atypia/follicular lesion of undetermined significance; BMI: body-mass index; FT3:free triiodothyroxine; FT4: free tetraiodothyroxine; TSH: thyroid stimulating hormone; TPOAb: anti-thyroid peroxidase antibody; TGAb: anti-thyroglobulin antibody; TC = total cholesterol; TG = triglyceride; HDL = high density lipoprotein; LDL = low density lipoprotein.

### US characteristics

Restricted with inclusion criteria, the associations between echo structures (solid/cystic/mixed), echogenicity (hypoechogenicity/isoechogenicity/hyperechogenicity) and malignancy risk were not explored in the study. Univariate analysis found that microcalcification, taller than wide shape and poorly-defined margin were associated with malignancy (Table 2).

**Table 2:** Conventional US characteristics of benign nodules and malignant nodules.

Variable	All patients (n = 153, %)	Malignancy (n, %)	Benign (n, %)	p value
**Microcalcification**				
Yes	40 (26.14)	28 (40.58)	12 (14.29)	<0.001
No	113 (73.86)	41 (59.42)	72 (85.71)	
**Taller than wide**				
Yes	61 (39.87)	40 (57.97)	21 (25.00)	<0.001
No	92 (60.13)	29 (42.03)	63 (75.00)	
**Margins**				
Well-defned	103 (67.32)	40 (57.97)	63 (75.00)	0.025
Poorly-defined	50 (32.68)	29 (42.03)	21 (25.00)	
**Echogenicity**				
Homogenicity	32 (20.92)	12 (17.39)	20 (23.81)	0.331
Inhomogenicity	121 (79.08)	57 (82.61)	64 (76.19)	
**High vascularity**				
Yes	63 (41.18)	29 (42.03)	34 (40.48)	0.236
No	90 (58.82)	40 (57.97)	50 (59.52)	

### Repeat FNA

Repeat FNA was performed in 47 (30.72%) TNs with initial FNA diagnosis as AUS/FLUS, and the second cytological diagnosis were: 9 (19.15%) unsatisfactory, 8 (17.02%) benign, 21 (44.68%) AUS/FLUS, 1 (2.1%) sFN, 5 (10.64%) sM, and 3 (6.38%) malignancy. In 47 repeat FNA, the rate of definite diagnosis (benign category and malignancy category included) was 11 (23.40%).

In 47 repeat FNA, the subsequent surgical specimens confirmed 18 (38.30%) malignant and 29 (61.70%) benign nodules. The 18 final malignant diagnosis was made in 2 (22.22%) unsatisfactory, 8 (36.36%) AUS/FLUS, 5 (100.00%) sM, and 3 (100.00%) malignancy; and benign diagnosis was made in 9 (77.78%) unsatisfactory, 8 (100.00%) benign and 1 (100.00%) sFN. There was no significant difference between the group with repeat FNA (38.30%) and the group without repeat FNA (48.11%), p = 0.260.

### Logistic regression analysis of risk factors in subcentimeter nodules

Multivariate logistic regression analysis found patient’s age, microcalcifcation, and taller than wide shape were independent risk factors for malignancy (Table 3). Gender, poorly-defined margin, which were found to be significant risk factors for malignancy in univariate analysis were not d to be significant and independent risk factors on multivariate analysis.

**Table 3:** Multivariate logistic regression analysis for malignancy in the thyroid nodule with AUS/FLUS.

Variable	B	S.E.	Wals	df	Sig.	Exp (B)	95%CI for Exp (B)	
							Lower	Upper
Age	1.103	0.472	5.477	1	0.019	3.015	1.196	7.596
Gender	−0.265	0.557	0.226	1	0.635	0.767	0.257	2.287
Taller than wide	2.378	0.493	*23*.*314*	1	0.000	10.785	4.108	28.319
Microcalcification	2.215	0.516	18.421	1	0.000	9.162	3.332	25.916
Poorly-defined margin	−0.800	0.424	3.556	1	0.059	0.450	0.196	1.032

### The malignancy risk on different subgroup

According to the presence or absence of the above three predictive factors, 153 patients could be classified into eight groups (Table 4). The highest rate of malignancy (8/8, 100%) was found in the group with three predictive factors, followed by 69.2–72.2% in the group with two predictive factors, then 13.6–33.3% in the group with one predictive factor, and the lowest was (3/16, 18.8%) in the group without any predictive factor. When patients with two or three predictive factors were classified into one group, patients with one or none of predictive factor were classified into the other group, there was a significant difference on malignancy between two groups (Table 5).

**Table 4:** The rates of malignancy in different groups stratified by three independent predictive factors.

Group	Definition	Case number (n)	Malignancy (n)	Benign (n)	Malignancy rate (%)
1	A + B + C	8	8	0	100.0
2	A + B	36	26	10	72.2
3	A + C	26	18	8	69.2
4	B + C	0	0	0	—
5	A	44	6	38	13.6
6	B	17	6	11	35.3
7	C	6	2	4	33.3
8	None	16	3	13	18.8%

A: stands for age < 55 year old; B: stands for taller than wide; C: stands for microcalcification.

**Table 5:** Comparison of malignancy rate between subgroups with different number of predictive factors.

Subgroup	Malignancy (n)	Benign (n)	p value	Hazard ratio	95%CI
Group with two or three predictors	52	18	<0.001	11.216	5.266–23.885
Group with one or none of predictors	17	66			

## Discussion

The overall AUS/FLUS utilization rate is 6.34% (324/5109) in our study, which is within the recommended 7% utilization for this diagnostic category. It is implied that there was a cancer risk range from 5% to 15% for this category. However, in the present study, the malignancy rate is 45.10%, which is much higher than the acceptable threshold. The characteristics of TNs in our study are specific, which may contribute to this result. Firstly, the included TNs are subcentimeter in size, which is always evaluated in China if there is any suspicious US characteristic. Small size of TNs decreases the satisfaction and accuracy of FNA, which may increase the inconclusive diagnosis especially unsatisfactory and AUS/FLUS category. Secondly and the most importantly, besides hypoechogenicity and solid characteristics, the included TNs also have one or more following suspicious US characteristics: microcalcification, poorly-defined margins, taller than wide shape. That is to say, the TNs in our study contain three or more suspicious characteristics, and should be categorized as TI-RADS 4b, 4c even 5. Therefore, the nodules in the current study should have greater risk of malignancy irrespective of small size.

For AUS/FLUS category, the common recommendation in ATA is to repeat US-FNA. Kuru B. *et al*.^16^ observed a significantly increasing malignicancy rate in patients with AUS/FLUS category when a repeat FNAB was performed compared with those without repeat FNAB. However, in other studies, there was no significant difference between patients with repeat FNAB and without repeat FNAB^17,18^. We found the similar result, which showed that the malignancy rate was 38.30% (18/47) in patients with repeat FNAB, while 48.11% (51/106) in patients without repeat FNAB. Hong SH *et al*.^18^ also did not find a significant difference in patients who had one AUS diagnosis and two successive AUS diagnosis. Repeat FNA may make a conclusive diagnosis in a proportion of cases^16^, therefore, it could be a reasonable choice for initial AUS diagnosis.

However, we found the conclusive diagnosis could be determined only in 23.40% (11/47) patients, including 3malignancy diagnosis in repeat FNA and 8 benign diagnosis. In the remaining 36 inconclusive diagnosis after repeat FNA, there were 2 (22.22%) patients with unsatisfactory diagnosis and 8 (36.36%) patients with AUS/FLUS, 5 (100.00%) patients with sM were finally determined to be malignancy after surgery. That is to say, the TNs diagnosed with malignancy and sM after repeat FNA should be surgically removed, and there was still a malignancy risk of 22.22–36.36% in unsatisfactory and AUS/FLUS category.

We find that younger age, microcalcification and taller than wide shape are three independent predictors for malignancy. Age is determined as one of prognostic factors for differentiate thyroid carcinoma (DTC), and the most important parameter in TNM staging system for DTC. However, little attention was made previously to its influence on the risk of malignancy. Recently, the eighth (8^th^) American Joint Commission on Cancer (AJCC) staging system recommended 55-year age as an ideal cut-off threshold for thyroid cancer staging^19^. In our study, we stratify the included patients into two groups: younger than 55-year age group and 55-year age or older group. For patients with AUS/FLUS, the rate of malignancy is 50.9% in the younger group, and 28.2% in the older group, which suggests that TN with an AUS/FLUS diagnosis in younger people is associated with a higher risk of malignancy when compared with older people. Todorovic, E. *et al*.^20^ also found that age <55 was a risk factor for malignancy in thyroid nodule with AUS/FLUS.

Ultrasound characteristics are always used to estimate the malignancy risk for thyroid nodules, and well-accepted worldwidely ultrasound characteristics for malignancy include solid structure, hypoechogenicity, irregular margins, microcalcification, and taller than wide shape, which are the foundations of TI-RADS. In the present study, for subcentimeter TNs with AUS/FLUS category, microcalcification and taller than wide shape are associated with the higher risk of malignancy. Chng. *et al*.^21^ and Maia. *et al*.^22^ reported that irregular margin of the TN was the utmost predictor for malignancy. However, we could only observe a tendency for association between irregular margin of TN and the risk of malignancy. The predictive values of solid structure and hypoechogenicity of nodule are not assessed in this study, because the included nodules are solid and hypoechoic TNs.

The highlights of the study should be acknowledged. Firstly, it is the first study with regard to the predictors for malignancy in subcentimeter TNs. The management of subcentimeter TNs is challenging, because widely-used TI-RADS and BSRTC after FNA are performed to estimate malignancy risk for the nodules ≥1 cm. Malignancy risk evaluation for subcentimeter TNs is rarely done in practice, because TN <1 cm is not routinely recommended for FNA in ATA guideline^10^.

Additionally, the decreased FNA accuracy in lower size nodules also aggravates the difficulty. The findings in our study would provide some evidence for malignancy risk evaluation in subcentimeter TNs. Secondly, microcalcification and taller than wide shape are found the more powerful predictive factors than patient’s age, which indicates that physicians should pay more attention to suspicious US characteristics than patient’s age when malignancy risk of thyroid nodule is assessed. Thirdly and the most importantly, we could stratify the subcentimeter TNs into two groups with different risk of malignancy: patients with two or more predictors have a 74.29% of malignancy risk, while the patients with one or none of predictor only have a 20.48% of malignancy risk. Our findings suggest that for subcentimeter TN with two or more predictors, even if the initial FNA diagnosis of AUS/FLUS, thyroidectomy should be a suitable choice; while for that with one or none of above predictor, repeat FNA or observation sounds reasonable.

There are several limitations in present study. Firstly, retrospective nature, single-institutional and small-sized characteristics may create a sampling bias. Secondly, to calculate a thorough risk of malignancy with AUS/FLUS category, all nodules should be removed and pathologically diagnosed, however, it is not possible in clinical settings because more than half of patients choose a conservative strategy. This leads to the selection bias in our study. Thirdly, molecular mutation test, particularly BRAF^V600E^ mutation was reported to be helpful for evaluating the malignancy risk and better decision-making^23–26^, however, it was not routinely available and could not be testified in our study. Finally, hypoechogenicity and solid composition were determined as independent predictors for malignancy in thyroid nodules larger than 1 cm, however, nodules in the current study are solid and hypoechoic characterized due to special inclusion criteria, we could not testify their predictive values for malignancy risk in subcentimeter thyroid nodules. Therefore, more large-scale, multicenter, prospective studies are required to define and verify accurate risk stratification and improve clinical management.

In summary, the risk of malignancy in the nodules with cytological diagnosis of AUS/FLUS in our study is higher than estimated by TBSRTC. Age younger than 55, taller than wide shape and microcalcification are determined as independent predictors for malignancy, and patients with two or more above predictors have a much higher risk of malignancy than those with one or none, which suggests surgical procedure may be a reasonable choice for the former while conservative strategy may be suitable for the latter.
